# Tacrolimus promotes hepatocellular carcinoma and enhances CXCR4/SDF-1α expression *in vivo*

**DOI:** 10.3892/mmr.2014.2302

**Published:** 2014-06-05

**Authors:** HUAQI ZHU, QIMAN SUN, CHANGJUN TAN, MIN XU, ZHI DAI, ZHENG WANG, JIA FAN, JIAN ZHOU

**Affiliations:** 1 Liver Cancer Institute, Zhongshan Hospital, Fudan University, Shanghai 200032, P.R. China; 2 Key Laboratory of Carcinogenesis and Cancer Invasion, Ministry of Education, Fudan University, Shanghai 200032, P.R. China; 3 Shanghai Key Laboratory of Organ Transplantation, Zhongshan Hospital, Fudan University, Shanghai 200032, P.R. China

**Keywords:** hepatocellular carcinoma, tacrolimus, CXCR4/SDF-1α, AMD3100

## Abstract

The aim of our study was to elucidate the effect of tacrolimus (FK506) and of C-X-C chemokine receptor type 4 (CXCR4), which is a receptor specific to the stromal cell-derived factor-1α (SDF-1α), on growth and metastasis of hepatocellular carcinoma (HCC). Following treatment with different concentrations of FK506, AMD3100 or normal saline (NS), the proliferation of Morris rat hepatoma 3924A (MH3924A) cells was measured by the MTT assay, the expression of CXCR4 was analyzed with immunohistochemistry, and the morphological changes and the invasiveness of cells were studied with a transwell assay and under a scanning electron microscope, respectively. In addition, August Copenhagen Irish rat models implanted with tumor were used to examine the pathological changes and invasiveness of tumor *in vivo*, the expression of CXCR4 in tumor tissues and the expression of SDF-1α in the adjacent tissues to the HCC ones, using immunohistochemistry. *In vitro*, FK506 (100–1,000 μg/l) significantly promoted the proliferation of MH3924A cells (P<0.01), and increased the expression of CXCR4 in MH3924A cells, albeit with no significance (P>0.05). By contrast, AMD3100 had no effect on the proliferation of MH3924A cells, but significantly reduced the expression of CXCR4 (P<0.05). The invasiveness of MH3924A cells was significantly (P<0.01) enhanced following treatment with FK506, SDF-1α, FK506 + AMD3100, FK506 + SDF-1α or FK506 + AMD3100 + SDF-1α. *In vivo*, tumor weight (P=0.041), lymph node metastasis (P=0.002), the number of pulmonary nodules (P=0.012), the expression of CXCR4 in tumor tissues (P=0.048) and that of SDF-1α in adjacent tissues (P=0.026) were significantly different between the FK506-treated and the NS group. Our results suggest that FK506 promotes the proliferation of MH3924A cells and the expression of CXCR4 and SDF-1α *in vivo*. Therefore, inhibiting the formation of the CXCR4/SDF-1α complex may partly reduce the promoting effect of FK506 on HCC.

## Introduction

Hepatocellular carcinoma (HCC) is one of the most prevalent tumor types (1). HCC represents a major histological subtype, accounting for 70–85% of total primary liver cancers worldwide (2), and is the third most common cause of cancer-related mortality (3). Hepatocarcinogenesis is a generally slow process, with initial genomic changes progressively altering the hepatocellular phenotype to produce cellular intermediates that evolve into HCC (4). HCC has a number of interesting epidemiologic features, such as dynamic temporal changes and marked variations among geographic regions and between genders. China alone accounts for >50% of the HCC cases worldwide (5).

Orthotopic liver transplantation (OLT) is a rational therapeutic option for patients with HCC (6), but recurrence still remains the main cause of death in HCC patients subjected to OLT (7). To reduce recurrence, an increasing number of physicians focus on strict selection criteria for transplantation candidates on the basis of tumor features (8). Tumor recurrence or metastasis is still an important and unresolved issue (9). Pharmacologic immunosuppression, required after transplantation, can accelerate tumor growth, although the effects of different immunosuppressive agents and regimes on HCC recurrence following OLT remain unclear (10).

Tacrolimus (FK506), a nonsteroidal topical immunomodulator, is an immunosuppressive drug widely used in patients subjected to organ transplantation (11,12). FK506 is believed to exert its immunosuppressive effect through targeted binding and inactivation of calcineurin (13). In previous studies, FK506 was reported to promote recurrence following lung transplantation (14), to enhance the invasive potential of HCC cells and to promote lymphatic metastasis (15). However in another study, FK506 did not promote the proliferation of rat HCC cells (16). Thus, the effects of FK506 on proliferation of cancer cells are still unclear.

Chemokines are soluble proteins of low molecular mass (5–15 kDa) that can mediate their corresponding effects by binding to specific, seven-transmembrane domain, G protein-coupled receptors (17). Chemokines and their receptors mediate the recruitment of immune cells in a number of diseases. In different tumor types, expression of the C-X-C chemokine receptor type 4 (CXCR4) has been associated with tumor dissemination and poor prognosis (18,19). Most particularly, the protein complex CXCR4/stromal cell-derived factor-1α (SDF-1α) is considered to play a central role in breast carcinoma metastasis (20), and might be involved in regulation of the metastatic behavior of tumor cells (21), as well as in metastasis of CXCR4^+^ tumor cells into the bone marrow and lymph nodes (22). However, whether the CXCR4/SDF-1α complex and downstream signaling has an effect on HCC remains uncertain.

In this context, the aim of the present study was to investigate the potential effects of FK506 and AMD3100, which is the antagonist of CXCR4, on the Morris rat hepatoma cell line MH3924A. Briefly, the proliferation, morphological changes and invasiveness of MH3924A cells, as well as the expression of CXCR4 in these cells, were examined *in vitro*. In addition, the growth and invasiveness of an implanted tumor, the expression of CXCR4 in tumor tissues and the expression of SDF-1α in the adjacent tissues to the HCC ones, were examined *in vivo*, in August Copenhagen Irish rats.

## Materials and methods

### Cell culture

Morris rat hepatoma 3924A cells (MH3924A) were purchased from the Institut für angewandte Zellkultur, (Munich, Germany). Cell lines were cultured in Dulbecco’s modified Eagle’s medium (DMEM), with 10% heat-inactivated fetal bovine serum (FBS; Invitrogen Life Technologies, Carlsbad, CA, USA) at 37°C in a humidified incubator with an atmosphere of 5% CO_2_. When the fusion rate reached >80%, the cells were digested with 25% trypsin and serial passages were performed at a dilution rate of 1:3.

### MTT assay

Tumor cell proliferation was determined by the 3-(4,5-dimethylthiazol-2-yl)-2,5-diphenyltetrazolium bromide assay (MTT; Sigma-Aldrich, St. Louis, MO, USA). Briefly, after MH3924A cells had reached the logarithmic growth phase, a 0.2-ml cell suspension at 1×10^4^ cells/ml was added into each well of a 96-well plate and cultured in DMEM with 10% FBS, 10 μg/l vascular endothelial growth factor and 0.1 g/l heparin for 24 h. When adherent growth was established, different concentrations of FK506 (10, 100 and 1,000 μg/l), AMD3100 (10, 50 and 100 μg/l) and FK506 (0 and 100 μg/l) + AMD3100 (0, 10, 50 and 100 μg/l) were added into the plates. Untreated cells cultured in medium alone were used as controls. After culturing for 48 h, 10 μl MTT (5 g/l) were added, and each well was incubated for 6 h; next, 150 μl/well dimethyl sulfoxide were added, followed by measurements of the absorbance at 570 mm on a spectrophotometer reader (Dynatech MRX, Elvetec, Genas, France). Each well was measured three times, and each sample was assayed in triplicate.

### Immunohistochemical assay and scanning electron microscopy (SEM)

MH3924A cells were cultured on sterile cover glasses (8×8 mm^2^) for 1 day, then cultured in DMEM with 100 μg/l FK506, or 50 μg/l AMD3100, or 100 μg/l FK506 + 50 μg/l AMD3100. When the cover glasses were covered with cells, the morphologic changes of MH3924A cells were observed with SEM, while a number of cover glasses were used for the immunohistochemical assay to determine the expression of CXCR4. This assay was performed using the anti-CXCR4 antibody (1:100 dilution; Wuhan Boshide Biotechnology Co., Wuhan, China) as the primary antibody and a horseradish peroxidase-conjugated secondary antibody (1:100; DakoCytomation, Glostrup, Denmark). The formed immunocomplex was visualized using the 3,3′-diaminobenzidine (DAB) reagent. The level of CXCR4 was quantified by a computer-assisted image system, which included a Leica CCD camera DFC420 connected to a Leica DM IRE2 microscope (Leica Microsystems, Wetzlar, Germany) (23,24). Image analysis yielded integrated optical density (IOD) values.

### Transwell invasion assay

Invasion assays were performed in 8-μm, 24-well, BioCoat Matrigel invasion chambers (Corning, Cambridge, MA, USA). Briefly, 1×10^4^ cells/ml were suspended in serum-free medium, and 50 μl of the suspension were added to each chamber, while 500 μl of the culture medium containing different concentrations and combinations of FK506, AMD3100 and SDF-1α (Table I) were added to the bottom chamber. Cells were allowed to invade for 12 h at 37°C in a humidified incubator with an atmosphere of 5% CO_2_; the cells that migrated to the top chamber were stained with Giemsa (Sigma-Aldrich) and were counted at a ×400 magnification under an electron microscope (Olympus, Tokyo, Japan). Assays were performed 3 times using triplicate wells.

### Rat model of liver tumor

Experiments were performed in 16 healthy August Copenhagen Irish rats (male, 16–20 weeks, weighing 240–300 g). The animals were handled in accordance with the regulations for laboratory animals. All animal experiments were in accordance with national guidelines and approved by the ethical committee of Zhongshan Hospital (Shanghai, China). The rat model of liver tumor was established as follows: First, MH3924A cells were collected and injected into the alar skin of rats. The tumors were removed from alar skin when grown to 2×1×1 mm^3^, and intrahepatic tumor implantation of rats was performed under aseptic conditions as described previously (25,26). Five days later, rats were randomly divided into two groups: one group was subcutaneously injected with normal saline for 14 days (NS group, n=8, 3 mg/kg/day), and the second group was subcutaneously injected with FK506 for 14 days (FK506 group, n=8, 0.3 mg/kg/day). Forty days following implantation, rats were sacrificed, and the weight of tumor, the volume of the fluid in the ascites, the incidence of lymphatic metastasis in the abdominal cavity and of abdominal wall metastasis were measured. In addition, the lungs were irrigated with 15% Indian ink, followed by counting of the number of metastatic nodules in the lung. The tumor and adjacent tissues, as well as healthy liver tissues, were harvested and preserved in 4% formalin for later use.

### Immunohistochemical assay

The tumor and adjacent tissues along with healthy liver tissues were sectioned (4 μm) according to the EliVision method (27). First, all sections were dried at 92°C for 30 min, treated with a 100% dimethylbenzene, 3% formalin-H_2_O_2_ solution, and washed with phosphate-buffered saline. Second, the sections were incubated with polyclonal antibodies of CXCR4 and SDF-1α purchased from Santa Cruz Biotechnology, Inc. (Santa Cruz, CA, USA) and Boster Biotechnology (Wuhan Boster Biological Technology, Ltd., Wuhan, Hubei, China), respectively, colored with DAB reagent and counterstained with hematoxylin and eosin. Following washing in PBS and drying and mounting in buffered glycerol-saline (90% glycerol, 10% PBS), the sections were observed under an inverted confocal microscope (DMRIE2; Leica Microsystems, Wetzlar, Germany). Sections were observed at a ×500 magnification, and 5 pictures were randomly selected and saved. The analysis of these images yielded IOD values.

### Statistical analysis

Data with a normal distribution are expressed as mean ± standard deviation (SD), while median values are used to represent data that were not normally distributed. We used the Windows version of the SPSS 16.0 software (SPSS, Inc., Chicago, IL, USA) to perform one-way analysis of variance for the comparison of normally distributed data, and the Mann-Whitney rank sum test implemented in the Image-Pro Plus (IPP) v6.0 software (Media Cybernetics, Inc., Bethesda, MD, USA) to compare the data that were not normally distributed, as for example the IOD values from the immunohistochemical assay. P<0.05 and P<0.01 were considered to indicate statistically significant differences.

## Results

### MTT assay

As shown by the MTT assay (Table II), treatment with a low concentration of FK506 (10 μg/l) did not significantly affect the proliferation of MH3924A cells (P=0.135). Upon treatment with higher concentrations of FK506 (100–1,000 μg/l), the proliferation of MH3924A cells was significantly enhanced (P<0.01). Treatment with AMD3100 at any concentration (10, 50 or 100 μg/l), had no obvious effect on MH3924A cell proliferation (P>0.05). However, when different concentrations of AMD3100 were combined with 100 μg/l FK506, the *in vitro* proliferation of MH3924A cells was increased (P<0.01, Table III). These data suggested that FK506 (≥100 μg/l) can promote the proliferation of MH3924A cells and that AMD3100 has no effect on the FK506-induced increase in proliferation.

### CXCR4 expression

**Figure 1:**
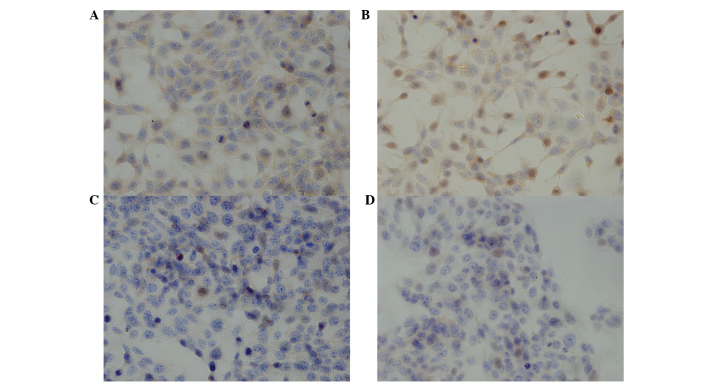
Immunohistochemical detection of the C-X-C chemokine receptor type 4 (CXCR4) protein in Morris rat hepatoma 3924A (MH3924A) cells (magnification, ×400). (A) Non-treated cells; (B) cells treated with 100 μg/l tacrolimus (FK506); (C) cells treated with 50 μg/l AMD3100; and (D) cells treated with 100 μg/l FK506 and 50 μg/l AMD3100. Yellow- and brown-color particles represent positive expression of CXCR4.

The effects of FK506 and AMD3100 on the expression of CXCR4 were studied in MH3924A cells with an immunohistochemical assay (Fig. 1). In non-treated MH3924A cells (Fig. 1A), CXCR4 expression was detectable at medium levels. CXCR4-stained particles were mainly distributed in the cytoplasm and the extracellular matrix, were occasionally present on the cytomembrane, and were absent from the nucleus. Following treatment with 100 μg/l FK506 (Fig. 1B), stronger staining of CXCR4 was observed, although this increase was not significant (P>0.05). By contrast, upon treatment with 50 μg/l AMD3100 (Fig. 1C), no or weaker expression of CXCR4 was observed compared to non-treated cells (P<0.05). The expression of CXCR4 was also negative or weaker in MH3924A cells treated with 100 μg/l FK506 combined with 50 μg/l AMD3100, compared to non-treated cells (P<0.05). These results suggested that the expression of CXCR4 is marginally increased by treatment with 100 μg/l FK506, but decreased by treatment with AMD3100 or FK506 + AMD3100.

### SEM

**Figure 2:**
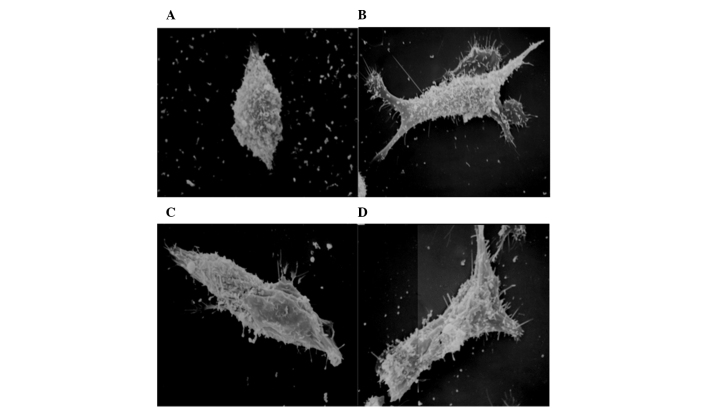
Scanning electron microscope images of Morris rat hepatoma 3924A (MH3924A) cells (magnification, ×500). (A) Non-treated cells; (B) cells treated with 100 μg/l tacrolimus (FK506); (C) cells treated with 50 μg/l AMD3100; and (D) cells treated with 100 μg/l FK506 and 50 μg/l AMD3100.

Examination of the cell morphology was performed with SEM (Fig. 2). The shape of non-treated MH3924A cells was regularly elliptical, and their surface was covered with microvilli and a few immobile processes (Fig. 2A). The MH3924A cells that were treated with 100 μg/l FK506 displayed a higher number of microvilli, longer immobile processes, and their shape was irregularly expanded compared to non-treated cells (Fig. 2B), suggesting stronger invasive ability. The cells treated with 50 μg/l AMD3100 were regularly elliptical similar to non-treated cells, while the number of microvilli and immobile processes did not significantly change (Fig. 2C). In addition, the morphology of MH3924A cells that were treated with 100 μg/l FK506 and 50 μg/l AMD3100 (Fig. 2D), was similar to that of cells treated with 100 μg/l FK506, displaying a higher number of microvilli and immobile processes, and irregular expansions; this morphology indicates increased cell invasiveness.

### Invasion assay

**Figure 3:**
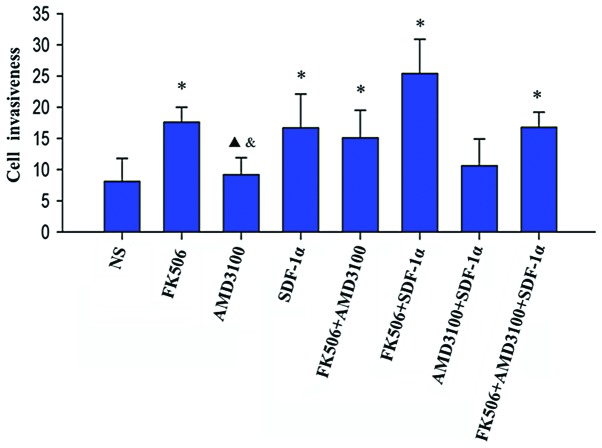
Changes in the invasiveness of Morris rat hepatoma 3924A (MH3924A) cells following treatment with different agents. The horizontal axis depicts the eight groups of cells depending on the treatment, and the vertical axis represents the invasiveness of MH3924A cells, as assessed by the transwell invasion assay. ^*^P<0.01, compared to the normal saline (NS) group; ^▲^P<0.05, compared to the tacrolimus (FK506) group; ^&^P<0.05, compared to the stromal cell-derived factor-1α (SDF-1α) group.

MH3924A cells are invasive. Therefore, their invasive ability was measured *in vitro* (Fig. 3). In the FK506 group, the number of invasive cells was significantly increased compared to the NS group (P<0.01). AMD3100 treatment had no significant effect on invasive ability compared to the NS group (P=0.09), but it clearly decreased the invasiveness of cells compared to treatment with FK506 (P=0.046) or SDF-1α (P=0.032), similarly to the NS group. In the SDF-1α group, the number of invasive cells was significantly increased compared to the NS group (P<0.01), but no significant difference was observed in the comparison with the FK506 group (P=0.881).

The number of invasive cells after treatment with the combination FK506 + AMD3100 was significantly increased compared to the NS group (P<0.01), but was not significantly affected compared to the FK506 (P=0.607) and SDF-1α (P=0.507) groups. In addition, the number of invasive cells was significantly increased in the FK506 + SDF-1α compared to the NS group (P<0.01), and was increased compared to the FK506 and the SDF-1α groups, but this increase was not significant (P=0.653 and P=0.548, respectively). Moreover, the number of invasive cells in the AMD3100 + SDF-1α group did not significantly change compared to the NS group (P=0.864). In the FK506 + AMD3100 + SDF-1α group, the invasive ability of MH3924A cells was significantly enhanced compared to the NS group (P<0.01), but not significantly enhanced in comparison to the FK506 (P=0.983) and the SDF-1α (P=0864) groups. These results showed that both FK506 and SDF-1α can enhance the invasive ability of MH3924A cells, and that AMD3100 can reduce the invasive ability of MH3924A cells when combined with SDF-1α.

### In vivo effects of tacrolimus

**Figure 4:**
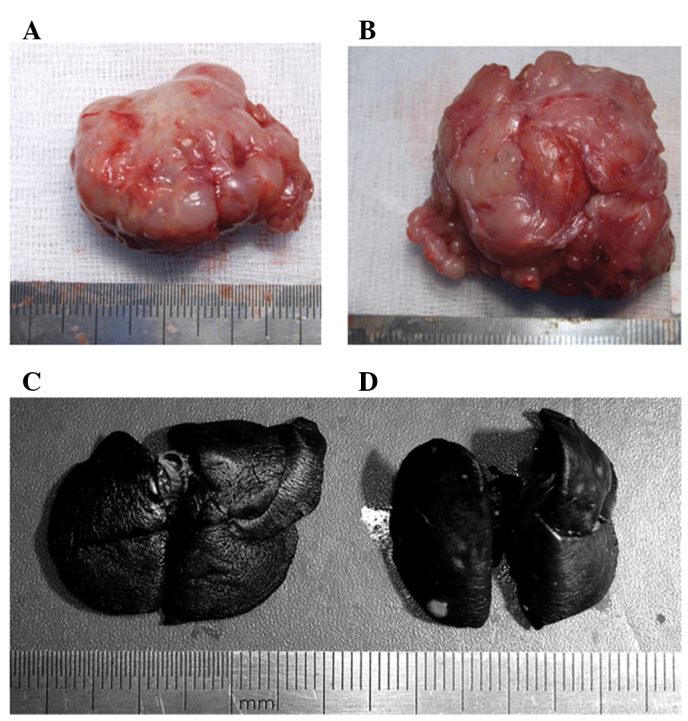
The tumor and the lung of rats treated with normal saline (NS) (3 mg/kg/day ×14 days) or tacrolimus (FK506) (0.3 mg/kg/day ×14 days), at 40 days following implantation. The tumor in the (A) NS; and (B) FK506 groups; the lung injected with Indian ink in the (C) NS; and (D) FK506 groups.

Following treatment with NS or FK506, none of the rats died or failed to keep the tumor implant until the day of observation. Tumor growth was examined after the implantation (Table IV). The liver of rats of the NS group was large, with an average weight at 15.56±11.17 g (Fig. 4A), and the liver of rats of the FK506 group was oversize, with an average weight at 28.19±3.89 g (Fig. 4B); no significant difference was found between the two groups (P=0.041). Regarding the number of metastatic nodules in the lungs, this was significantly increased in the FK506 compared to the NS group (6.50±4.63 vs. 1.39±1.25, P=0.012) (Fig. 4C–D). Moreover, the ascite fluid volume was increased in rats of the FK506 group compared to the NS group, although this change was not statistically significant (21.25±6.94 vs. 13.13±21.87 ml, P=0.317). The rate of lymph node metastasis, as well as the number of pulmonary nodules, were significantly increased in the FK506 compared to the NS group (P=0.002 and P=0.012, respectively). However, no significant difference in the rate of abdominal wall metastasis was observed between the two groups (P=0.442).

### Immunohistochemical detection of CXCR4 and SDF-1α

**Figure 5:**
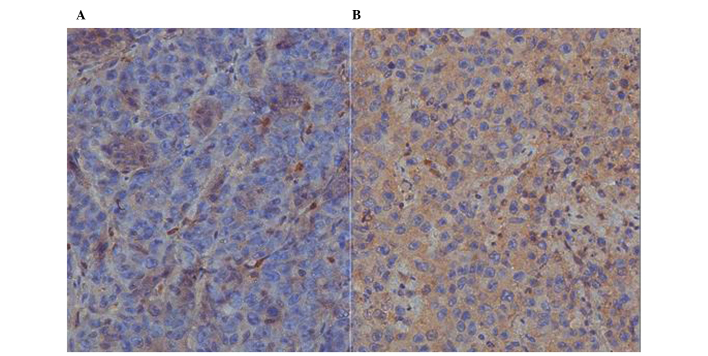
Immunohistochemical detection of the C-X-C chemokine receptor type 4 (CXCR4) protein in liver tissues of rats with hepatocellular carcinoma (magnification, ×400). Positive expression of CXCR4 in the (A) normal saline; and (B) tacrolimus groups is represented by brown-color particles.

**Figure 6:**
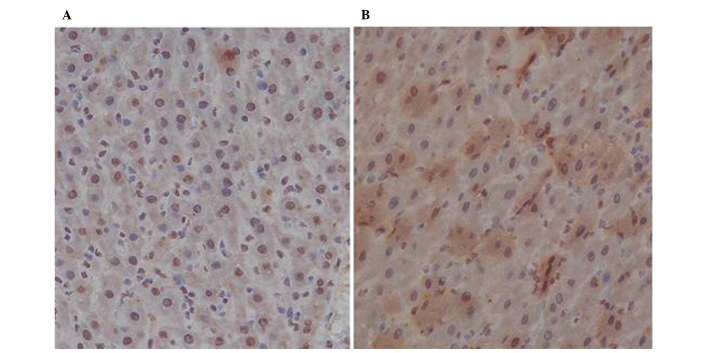
Immunohistochemical detection of the stromal cell-derived factor-1α (SDF-1α) protein in the adjacent tissues of rats with hepatocellular carcinoma (magnification, ×400). Positive expression of SDF-1α in the (A) normal saline; and (B) tacrolimus groups is represented by brown-color particles.

Immunohistochemical staining was performed in order to detect the expression of CXCR4 in tumor HCC tissues and of SDF-1α in the tissues adjacent to the HCC ones (Table IV). In the NS group, CXCR4 was expressed at medium levels, with an average IOD at 1.48±0.29 (Fig. 5A), but in the FK506 group, the expression of CXCR4 was higher, with an average IOD at 2.50±0.62 (Fig. 5B). The increased expression of CXCR4 following FK506 treatment was significant (P=0.048). In addition, the expression level of SDF-1α in tissues adjacent to the HCC ones was medium in the NS group, with an average IOD at 1.46±0.39 (Fig. 6A). In the FK506 group (Fig. 6B), the expression of SDF-1α in the adjacent tissues was significantly higher compared to the NS group (P=0.026), with an average IOD at 2.54±0.94.

## Discussion

OLT is the best therapeutic option for patients with HCC. However, the pharmacologic immunosuppression regimes required at the post-transplantation stage can affect the recurrence of HCC by accelerating tumor growth, and the effects of immunosuppression on post-OLT HCC recurrence have been poorly investigated (8). Therefore, finding an effective method to reduce the recurrence of HCC while using immunosuppressive agents is an issue of great importance in the clinic.

In this study, we investigated the effects of the immunosuppressive drug tacrolimus (FK506) on the proliferation of MH3924A cells. The growth of MH3924A cells was significantly increased following treatment with 100 μg/l FK506, while 10 μg/l FK506 had no significant effect on cell growth, thus indicating that high concentrations of FK506 can promote the proliferation of MH3924A cells *in vitro*. Moreover, in an *in vivo* HCC rat model, the metastatic rate of tumor in the abdominal wall, the number of lymph nodes, as well as the lung size, were increased upon FK506 treatment compared to treatment with NS. This result was consistent with the *in vitro* experiments. Therefore, FK506 can promote the proliferation of MH3924A cells. Treatment with FK506 resulted in a dose-dependent increase in the number of pulmonary metastases in a previous study (28), and stimulated the Rho/ROCK signaling pathway to enhance the invasiveness of HCC (16). This is evidence that FK506 may promote the progress of HCC (29).

Moreover, the effect of the CXCR4/SDF-1α complex in HCC was investigated in the present study. The SDF-1α chemokine and its receptor were suggested to play an important role in metastasis towards lymph nodes in cervical cancer (30). In our study, CXCR4 was found to be expressed in MH3924A cells. When the cells were treated with FK506, the expression of CXCR4 did not significantly change, while treatment with FK506 and AMD3100 significantly decreased its expression. These results suggest that FK506 can not alter the expression of CXCR4 in MH3924A cells. Nevertheless, in our *in vivo* experiments, the expression of CXCR4 in tumor tissues, as well as the expression of SDF-1α in the tissues adjacent to the HCC ones, was increased following FK506 treatment. This might be due to the metastatic potential of MH3924A cells. In a previous study, the HCC cell line HepG2 was found to be unresponsive to SDF stimulation due to an unknown defect, which was identified to involve a step after receptor binding but before the activation of the signaling cascade (31). CXCR4 was also reported to be ‘trapped’ in the cytoplasm and not recruited to the cell surface in response to standard extrinsic stimuli in the majority of HCC cell lines, resulting in a negligible response to SDF-1 (32). Hence, these two proteins might be involved in the HCC invasion process, although their specific roles need to be further investigated.

The invasiveness of MH3924A cells was also studied *in vitro*. FK506 treatment significantly increased the invasiveness of MH3924A cells, while AMD3100 treatment decreased it. Treatment with SDF-1α also clearly enhanced the invasiveness of MH3924A cells, similar to FK506, and this increase was also observed for the treatment combining FK506 with AMD3100 and/or SDF-1α. By contrast, AMD3100 + SDF-1α treatment reduced cell invasiveness to levels comparable to those of the NS group. These results are in agreement with previous studies, reporting that the biological effects of SDF-1 are strongly inhibited by AMD3100 (33) and that AMD3100 can block HIV-1 entry via its antagonistic effect on CXCR4 (34). Similarly, our results indicated that both FK506 and SDF-1α enhance the invasiveness of MH3924A cells, and that AMD3100 attenuates the effects of FK506 by blocking CXCR4.

In conclusion, FK506 promotes the proliferation of MH3924A cells, increases the expression of CXCR4 in tumor tissues and that of SDF-1α in adjacent tissues to the HCC ones, enhances the invasiveness of MH3924A cells and significantly intensifies a number of pathological features. Moreover, SDF-1α increases, while AMD3100 decreases, the invasiveness of MH3924A cells *in vitro*, potentially by blocking the formation of the CXCR4/SDF-1α complex. Therefore, minimizing the use of FK506 may reduce the recurrence of OLT, and the CXCR4/SDF-1α interaction may play vital roles in HCC metastasis. However, whether CXCR4/SDF-1α can be used as a new target of prevention and treatment of OLT needs to be further investigated.

## Figures and Tables

**Table I:** The groups in the transwell invasion assay.

Group	Top chamber	Bottom chamber
Control	DMEM	DMEM
FK506	10 μg/l FK506	DMEM
AMD3100	10 μg/l AMD3100	DMEM
SDF-1α	DMEM	1 μg/l SDF-1α, 10 μg/l SDF-1α
FK506 +AMD3100	10 μg/l FK506, 10 μg/l AMD3100	DMEM
FK506+SDF-1α	10 μg/l FK506	10 μg/l SDF-1α
AMD3100 +SDF-1α	10 μg/l AMD3100	10 μg/l SDF-1α
FK506+AMD3100 +SDF-1α	10 μg/l FK506, 10 μg/l AMD3100	10 μg/l SDF-1α

DMEM, Dulbecco’s modified Eagle’s medium; FK506, tacrolimus; SDF-1α, stromal cell-derived factor-1α.

**Table II:** The absorbance values of MH3924A cells treated with different concentrations of FK506 or AMD3100.

Group	0 μg/l	10 μg/l	100 μg/l	1,000 μg/l
FK506				
Absorbance value	0.80±0.17	1.17±0.49	1.53±0.39	1.64±0.19
P	-	0.135	0.006^a^	0.002^a^
AMD3100				
Absorbance value	0.80±0.17	0.84±0.06	0.86±0.10	0.85±0.10
P	-	0.760	0.788	0.812

a P<0.01 compared to the 0 μg/l group.

MH3924A, Morris rat hepatoma 3924A cells; FK506, tacrolimus.

**Table III:** The absorbance values of MH3924A cells treated with different concentrations of FK506 combined with different concentrations of AMD3100.

AMD3100 (μg/l)	FK506		

	0 μg/l	100 μg/l	P
0	0.80±0.17	1.53±0.39	0.006^a^
10	0.84±0.06	1.79±0.10	0.000^a^
50	0.86±0.10	1.46±0.27	0.005^a^
100	0.85±0.10	1.55±0.31	0.002^a^

a P<0.01 compared to the 0 μg/l group.

MH3924A, Morris rat hepatoma 3924A cells; FK506, tacrolimus.

**Table IV:** Parameters of the *in vivo* HCC models following treatment with FK506 and NS.

Parameters	NS	FK506	P
Tumor weight (g)^a^	15.56±11.17	28.19±3.89	0.041^c^
Ascite fluid (ml)^a^	13.13±21.87	21.25±6.94	0.317
Lymph node metastasis^b^	0	8	0.002^c^
Abdominal wall metastasis^b^	2	5	0.442
Pulmonary nodules (n)^a^	1.39±1.25	6.50±4.63	0.012^c^
Expression of CXCR4^a^	1.48±0.29	2.50±0.62	0.048^c^
Expression of SDF-1α^a^	1.46±0.39	2.54±0.94	0.026^c^

a Normally distributed data, expressed as mean ± SD;

b distribution of data was not normal, median values shown;

c P<0.05, compared to the NS group.

HCC, hepatocellular carcinoma; NS, normal saline; FK506, tacrolimus; CXCR4, C-X-C chemokine receptor type 4; SDF-1α, stromal cell-derived factor-1α.
